# Domestic

**Published:** 1841-08-07

**Authors:** 


### DOMESTIC.

*Milk Sickness.*—The "*Mill Stone Knob*" in Sumner county, Tenn., is one of the localities which have been famed, since the settlement of the country, for giving rise to milk sickness. It is now generally called the "*poison* knob." Yet domestic animals of every description may be seen, at this season, feeding upon the grass which grows luxuriantly on its sides wherever the sun has gained admission to the soil, and it is understood that they may continue to range it with safety until about the commencement of winter. In a hasty excursion which we made to the knob, a few weeks since, we saw, every now and then, the spot where a fire had been kindled to consume the carcass of some animal which had perished of the poison. Such occurrences, we learnt, were still not uncommon in the neighbourhood, yet owing to the caution of the people in keeping their milch-cows upon pastures which have been cultivated, a case of poison in the human subject has not occurred for three years. The country around the knob is thickly settled, and families are found living on the sides of the hill. They feel safe so long as they abstain from the flesh and milk of animals that have been ranging the "poison knob," or in other words, while they confine their stock to their own cultivated grounds. This knob is one of the most fertile spots to be seen in that fertile region of country, and has a black, rich soil to its very summit, which is now covered with a luxuriant growth of cane. So fine is the pasture upon it, in winter, that persons drive their young cattle to it to pass that season. A few die of the poison, but they feel themselves indemnified in the saving of forage and labor that would be necessary to take the survivors through the winter. I need hardly add, that the cause of this singular disease has not been discovered. The *remedy* for it is *prevention*, and this consists in bringing the soil under the dominion of the plough. One year's cultivation effectually eradicates the poison, and forever afterwards the lands infected by it, if sowed in grass, may be depastured with impunity. It is a fact tested by all with whom I conversed, that hogs and buzzards, as well as hens and turkeys, are poisoned by the flesh of animals that have died from this cause. Dr. Graffe, it may be known to some of the readers of this Journal, came to a contrary conclusion after some experiments upon the hog, as related in a late number of the Medical Examiner.—*West. Jour. of Med. and Surg.*

---

*Fever in the West.* By M. L. Linton, M.D.—*Dear Sir,*—I shall, in the present communication, afford you a very brief account of the various forms of fever which have prevailed in this and the adjoining counties during the past autumn and winter—a task which I should not attempt, but for some highly important therapeutic facts, furnished me by Dr. Shuck, a very respectable practitioner of a neighbouring town. All the physicians, with whom I have conversed on the subject, (for I arrived at home too late to observe for myself,) represent the disease as congestive and adynamic from the beginning—rarely followed by much reaction—promptly fatal in a great many cases, and not yielding with facility to the ordinary modes of treatment in any. The winter diseases (which I have had the opportunity of observing) have been of the same type, and even pleurisy and other inflammatory affections of the chest have been best treated without bleeding or with very little. But to proceed to Dr. Shuck's communication. Speaking of the epidemic of last winter, he says, "The disease was ushered in by symptoms not apparently different from those of ordinary intermittent and remittent fever; and that tightness and tenderness of the epigastrium were rarely absent. We gave to it the general appellation of congestive fever, though there was every variety of type from the perfectly intermittent to the purely continued. Those cases in which the apyrexia was distinct, I treated successfully with quinine. The remittent and continued forms, I treated, at the commencement of the season, by bleeding, purging, and other antiphlogistic means common in such cases. This, as far as I know was the treatment pursued by all the members of the profession in this place, and all had equally bad success. I recollect no case of recovery in which free bleeding was resorted to, and but very few where large and repeated doses of calomel were given."

I became dissatisfied with our mode of treatment, and determined to use quinine in all cases, even the most decidedly continued. The first case to which I was called after coming to this resolution, was a robust and somewhat intemperate man about forty years of age. He had had fever several days when I first saw him—it was of the continued form—he had been bled the day previous to my visit, but without any relief. I immediately gave him 15 grs. of calomel with a little rhubarb: it acted, but the patient remained in the same condition. I then gave him quinine freely every hour during eight hours—then directed a second purge similar to the first—then the quinine as before. Before the expiration of the first eight hours the fever subsided, and a free perspiration supervened. Convalesence was rapid. After this I treated all my cases in a similar way, and *lost not a single one*, though I had a great number. I treated one hundred and fifty-three cases of the prevailing fever of last summer, and lost but one, and that was a black child that had been neglected in the commencement.

One occurrence I must narrate before I close. On the 10th of July last I was called to see Mr. B., a gentleman of middle age and delicate constitution. He told me that he had had a chill some days previous: and that his fever had continued from that time. He required me to bleed him, assuring me that he had always found bleeding and purging his best remedies under similar circumstances. I remonstrated with him, and gave him the history of my treatment of the disease; but he could not be convinced that he did not understand his own case better than I did, and was therefore inflexible in his demands for bleeding, &c. His pulse was full—skin hot—thirst great. I bled him to the amount of a pint—his pulse sunk, but he was not at all relieved—became restless and complained of great prostration. He then gave up his case into my hands; though on the next day he requested me to bleed him again, which I positively refused to do. I commenced by giving a dose of calomel followed by castor oil, which produced free bilious discharges. I then commenced with the quinine; but owing to the irritable condition of his stomach, it was given in small portions. His case was very protracted, but he ultimately recovered. On the same day that B. was attacked, another gentleman in his immediate vicinity was attacked with the same disease, alike in all the symptoms as described by several intelligent gentlemen who were friends of both the sick men, and saw them every day. This gentleman, Mr. M., was treated by a physician who adopted the depleting plan. He was bled on the same day that I bled Mr. B. He was then freely purged. The next day the bleeding was repeated, and the patient sunk rapidly, and died. I have not a shadow of doubt that such would have been the fate of Mr. B. had I repeated the bleeding; and I can unequivocally assert, that the cases in Lebanon and its vicinity, when treated by the depleting plan, generally terminated unfavourably; and that those which were treated with quinine recovered almost without an exception."

As my paper is almost exhausted, I shall close by rehearsing the Doctor's treatment in a few words. He commences generally with a dose of calomel and rhubarb, or an emeto-cathartic, by the action of which the contents of the bowels are evacuated—then gave the quinine every hour for about eight hours, in two or three grain doses—then used another purgative, and again resumed the quinine as before; sometimes the repetition of the course was unnecessary. In every case the quinine seems to have induced a pleasant perspiration. I have already said that the Doctor employed the above plan in the continued as well as the intermittent forms of the epidemic, and with similar success. In his communication to me he states, that the dysentery, which prevailed concomitantly, was equally amenable to the same course. I have no room for comment, nor do the foregoing facts need any. They show the necessity for close observation—especially *at the commencement* of epidemics. That course which cures the first three or four cases will, with slight variations and occasional exceptions, cure the rest, and vice versa—and thus a therapeutic rule is furnished of more practical utility, than any that could be arrived at by a priori reasoning on principles. This is not a new rule, to be sure, for I believe it was the father of medicine who said—*Naturam morborum curationes ostendunt.—Ibid.*

